# 4-Allyl­morpholin-4-ium bromide

**DOI:** 10.1107/S1600536812010793

**Published:** 2012-03-17

**Authors:** Meng Ting Han

**Affiliations:** a Ordered Matter Science Research Center, College of Chemistry and Chemical, Engineering, Southeast University, Nanjing 211189, People’s Republic of China

## Abstract

The title compound, C_7_H_14_NO^+^·Br^−^, was formed by reaction of 4-allyl­morpholine and hydrogen bromide. In the crystal, mol­ecules are connected *via* N—H⋯Br and C—H⋯Br hydrogen bonds, forming a three-dimensional network.

## Related literature

For selected sources of ferroelectric materials, see: Haertling (1999 ▶); Homes *et al.* (2001 ▶); Fu *et al.* (2009 ▶); Hang *et al.* (2009 ▶).
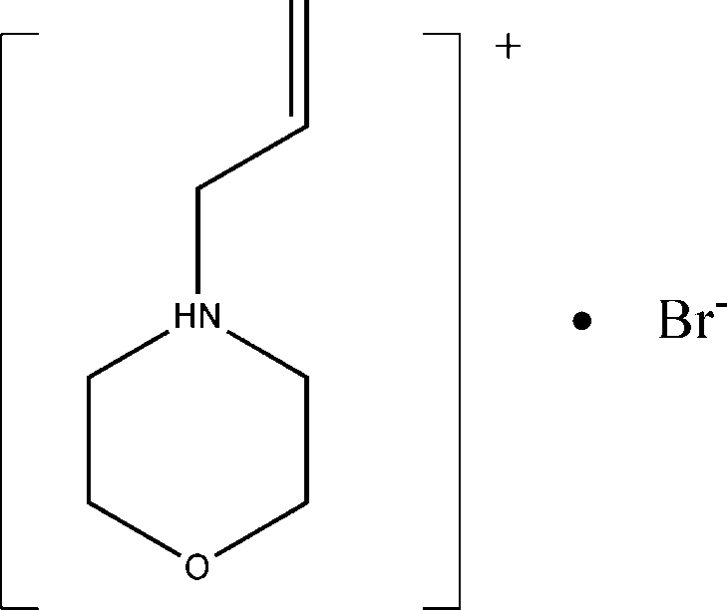


## Experimental

### 

#### Crystal data

C_7_H_14_NO^+^·Br^−^
*M* *_r_* = 208.10
Triclinic, 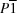

*a* = 7.4115 (15) Å
*b* = 7.9727 (16) Å
*c* = 8.7948 (18) Å
α = 66.43 (3)°
β = 82.14 (3)°
γ = 85.78 (3)°
*V* = 471.75 (17) Å^3^
*Z* = 2
Mo *K*α radiation
μ = 4.30 mm^−1^
*T* = 293 K
0.33 × 0.28 × 0.20 mm

#### Data collection

Rigaku SCXmini diffractometer
Absorption correction: multi-scan (*CrystalClear*; Rigaku, 2005 ▶) *T*_min_ = 0.252, *T*_max_ = 0.423
4897 measured reflections
2155 independent reflections
1786 reflections with *I* > 2σ(*I*)
*R*_int_ = 0.043
2 standard reflections every 150 reflections intensity decay: none

#### Refinement

*R*[*F* ^2^ > 2σ(*F* ^2^)] = 0.039
*wR*(*F* ^2^) = 0.099
*S* = 1.07
2155 reflections
92 parameters
H-atom parameters constrained
Δρ_max_ = 0.59 e Å^−3^
Δρ_min_ = −0.42 e Å^−3^

### 

Data collection: *CrystalClear* (Rigaku, 2005 ▶); cell refinement: *CrystalClear*; data reduction: *CrystalClear*; program(s) used to solve structure: *SHELXS97* (Sheldrick, 2008 ▶); program(s) used to refine structure: *SHELXL97* (Sheldrick, 2008 ▶); molecular graphics: *SHELXTL* (Sheldrick, 2008 ▶); software used to prepare material for publication: *SHELXTL*.

## Supplementary Material

Crystal structure: contains datablock(s) I, global. DOI: 10.1107/S1600536812010793/mw2056sup1.cif

Structure factors: contains datablock(s) I. DOI: 10.1107/S1600536812010793/mw2056Isup2.hkl

Supplementary material file. DOI: 10.1107/S1600536812010793/mw2056Isup3.cml

Additional supplementary materials:  crystallographic information; 3D view; checkCIF report

## Figures and Tables

**Table 1:** Hydrogen-bond geometry (Å, °)

*D*—H⋯*A*	*D*—H	H⋯*A*	*D*⋯*A*	*D*—H⋯*A*
N1—H1*C*⋯Br1^i^	0.91	2.31	3.218 (2)	175
C1—H1*A*⋯Br1^ii^	0.97	2.93	3.846 (4)	158
C5—H5*B*⋯Br1^iii^	0.97	2.86	3.796 (3)	162

Symmetry codes: (i) 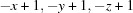; (ii) 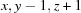; (iii) 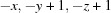.

**Fig. 1.**
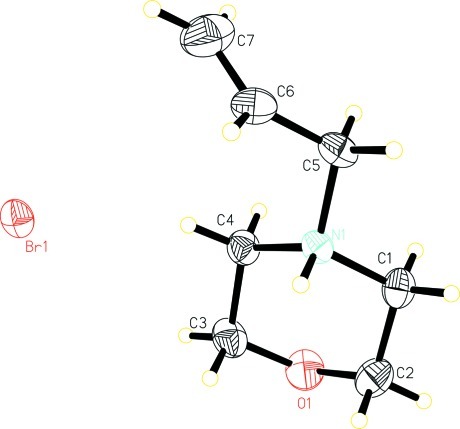
Perspective structure of the title compound, showing the atom-numbering scheme. Displacement ellipsoids are drawn at the 30% probability level.

**Fig. 2.**
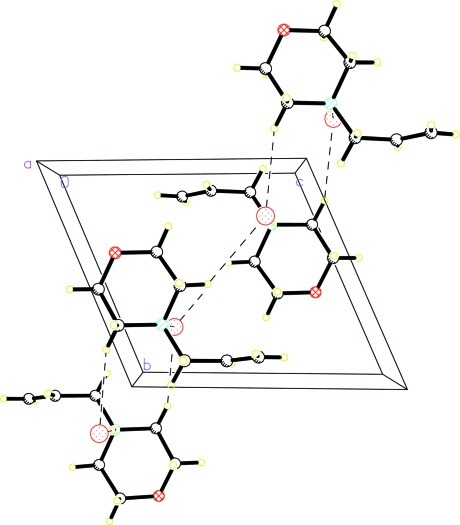
The crystal packing of the title compound viewed along the a axis showing the hydrogen bonding network. Some of the H-atoms have been ommitted for clarity.

## supplementary crystallographic information

### Comment

At present, much attention in ferroelectric material field is focused on developing ferroelectric pure organic or inorganic compounds (Haertling *et al.* 1999; Homes *et al.* 2001). Recently we have reported the synthesis of a variety of compounds (Fu *et al.*, 2009; Hang *et al.*, 2009), which have potential piezoelectric and ferroelectric properties. In order to find more dielectric ferroelectric materials, we investigate the physical properties of the title compound (Fig. 1). The dielectric constant of the title compound as a function of temperature indicates that the permittivity is basically temperature-independent (dielectric constant equaling to 0.6 to 1.42), suggesting that this compound should be not a real ferroelectrics or there may be no distinct phase transition occurred within the measured temperature range. Similarly, below the melting point (408 K) of the compound, the dielectric constant as a function of temperature also goes smoothly, and there is no dielectric anomaly observed (dielectric constant equaling to 0.6 to 1.42).Herein, we report the synthesis and crystal structure of the title compound.

As can be seen from the packing diagram (Fig. 2), molecules are connected *via* intermolecular N—H···Br and C—H···Br hydrogen bonds to form a three-dimensional network. Dipole–dipole and van der Waals interactions are also operative in organizing the molecular packing.

### Experimental

A mix of 4-allylmorpholine (0.762 g, 0.006 mol) and hydrogen bromide (1.212 g, 0.006 mol) in water (20 ml) was stirred until clear. After several days, the title compound was formed and recrystallized from solution to afford red prismatic crystals suitable for X-ray analysis.

### Refinement

H atoms were positioned geometrically and refined using a riding model, with C—H = 0.97 Å and *U*_iso_(H) = 1.2_eq_(C).

### Crystal data

**Table:**

C_7_H_14_NO^+^·Br^−^	*Z* = 2
*M**_r_* = 208.10	*F*(000) = 212
Triclinic, *P*1	*D*_x_ = 1.465 Mg m^−^^3^
Hall symbol: -P 1	Mo *K*α radiation, λ = 0.71073 Å
*a* = 7.4115 (15) Å	Cell parameters from 2158 reflections
*b* = 7.9727 (16) Å	θ = 2.3–27.5°
*c* = 8.7948 (18) Å	µ = 4.30 mm^−^^1^
α = 66.43 (3)°	*T* = 293 K
β = 82.14 (3)°	Prismatic, red
γ = 85.78 (3)°	0.33 × 0.28 × 0.20 mm
*V* = 471.75 (17) Å^3^	

### Data collection

**Table:**

Rigaku SCXmini diffractometer	1786 reflections with *I* > 2σ(*I*)
Radiation source: fine-focus sealed tube	*R*_int_ = 0.043
Graphite monochromator	θ_max_ = 27.5°, θ_min_ = 3.5°
ω scans	*h* = −9→9
Absorption correction: multi-scan (*CrystalClear*; Rigaku, 2005)	*k* = −10→10
*T*_min_ = 0.252, *T*_max_ = 0.423	*l* = −11→11
4897 measured reflections	2 standard reflections every 150 reflections
2155 independent reflections	intensity decay: none

### Refinement

**Table:**

Refinement on *F*^2^	Secondary atom site location: difference Fourier map
Least-squares matrix: full	Hydrogen site location: inferred from neighbouring sites
*R*[*F*^2^ > 2σ(*F*^2^)] = 0.039	H-atom parameters constrained
*wR*(*F*^2^) = 0.099	*w* = 1/[σ^2^(*F*_o_^2^) + (0.0469*P*)^2^ + 0.0113*P*] where *P* = (*F*_o_^2^ + 2*F*_c_^2^)/3
*S* = 1.07	(Δ/σ)_max_ = 0.001
2155 reflections	Δρ_max_ = 0.59 e Å^−^^3^
92 parameters	Δρ_min_ = −0.42 e Å^−^^3^
0 restraints	Extinction correction: *SHELXL97* (Sheldrick, 2008), Fc^*^=kFc[1+0.001xFc^2^λ^3^/sin(2θ)]^-1/4^
Primary atom site location: structure-invariant direct methods	Extinction coefficient: 0.193 (10)

### Special details

**Table:**

Geometry. All e.s.d.'s (except the e.s.d. in the dihedral angle between two l.s. planes) are estimated using the full covariance matrix. The cell e.s.d.'s are taken into account individually in the estimation of e.s.d.'s in distances, angles and torsion angles; correlations between e.s.d.'s in cell parameters are only used when they are defined by crystal symmetry. An approximate (isotropic) treatment of cell e.s.d.'s is used for estimating e.s.d.'s involving l.s. planes.
Refinement. Refinement of *F*^2^ against ALL reflections. The weighted *R*-factor *wR* and goodness of fit *S* are based on *F*^2^, conventional *R*-factors *R* are based on *F*, with *F* set to zero for negative *F*^2^. The threshold expression of *F*^2^ > σ(*F*^2^) is used only for calculating *R*-factors(gt) *etc*. and is not relevant to the choice of reflections for refinement. *R*-factors based on *F*^2^ are statistically about twice as large as those based on *F*, and *R*- factors based on ALL data will be even larger.

### Fractional atomic coordinates and isotropic or equivalent isotropic displacement parameters (Å^2^)

**Table:**

	*x*	*y*	*z*	*U*_iso_*/*U*_eq_	
O1	0.2009 (4)	0.5927 (3)	0.8606 (3)	0.0636 (7)	
N1	0.2630 (3)	0.2655 (3)	0.7998 (3)	0.0367 (5)	
H1C	0.3863	0.2567	0.7988	0.044*	
C1	0.1820 (5)	0.2637 (5)	0.9663 (4)	0.0509 (8)	
H1A	0.2179	0.1515	1.0548	0.061*	
H1B	0.0501	0.2684	0.9729	0.061*	
C2	0.2476 (6)	0.4268 (5)	0.9881 (5)	0.0620 (10)	
H2A	0.1941	0.4260	1.0955	0.074*	
H2B	0.3789	0.4176	0.9876	0.074*	
C3	0.2827 (5)	0.5971 (4)	0.7029 (4)	0.0573 (9)	
H3A	0.4141	0.5878	0.7018	0.069*	
H3B	0.2531	0.7132	0.6158	0.069*	
C4	0.2186 (4)	0.4436 (4)	0.6671 (4)	0.0468 (7)	
H4A	0.0880	0.4555	0.6626	0.056*	
H4B	0.2773	0.4495	0.5596	0.056*	
C5	0.2028 (4)	0.1047 (4)	0.7716 (4)	0.0468 (8)	
H5A	0.2321	−0.0079	0.8630	0.056*	
H5B	0.0718	0.1119	0.7706	0.056*	
C6	0.2918 (5)	0.1003 (5)	0.6127 (5)	0.0550 (9)	
H6A	0.4171	0.0794	0.6028	0.066*	
C7	0.2073 (8)	0.1237 (6)	0.4856 (6)	0.0848 (14)	
H7A	0.0820	0.1449	0.4911	0.102*	
H7B	0.2721	0.1192	0.3889	0.102*	
Br1	0.29920 (3)	0.76037 (4)	0.22722 (4)	0.0524 (2)	

### Atomic displacement parameters (Å^2^)

**Table:**

	*U*^11^	*U*^22^	*U*^33^	*U*^12^	*U*^13^	*U*^23^
O1	0.0796 (17)	0.0498 (14)	0.0661 (16)	0.0035 (12)	0.0026 (13)	−0.0323 (12)
N1	0.0285 (10)	0.0366 (13)	0.0429 (13)	0.0007 (9)	−0.0024 (9)	−0.0142 (10)
C1	0.0496 (17)	0.0539 (19)	0.0436 (18)	0.0032 (15)	0.0002 (14)	−0.0160 (15)
C2	0.070 (2)	0.072 (3)	0.051 (2)	0.001 (2)	−0.0022 (18)	−0.034 (2)
C3	0.070 (2)	0.0361 (17)	0.061 (2)	−0.0049 (16)	0.0035 (18)	−0.0178 (16)
C4	0.0537 (18)	0.0369 (16)	0.0455 (18)	−0.0008 (14)	−0.0055 (14)	−0.0121 (14)
C5	0.0414 (16)	0.0361 (16)	0.062 (2)	−0.0020 (13)	−0.0098 (14)	−0.0172 (15)
C6	0.0551 (19)	0.0498 (19)	0.069 (2)	0.0016 (16)	−0.0125 (17)	−0.0316 (18)
C7	0.110 (4)	0.075 (3)	0.086 (3)	0.007 (3)	−0.029 (3)	−0.044 (3)
Br1	0.0324 (2)	0.0604 (3)	0.0540 (3)	0.00297 (15)	−0.00786 (14)	−0.01133 (17)

### Geometric parameters (Å, º)

**Table:**

O1—C2	1.407 (4)	C3—H3A	0.9700
O1—C3	1.424 (4)	C3—H3B	0.9700
N1—C4	1.483 (3)	C4—H4A	0.9700
N1—C1	1.500 (4)	C4—H4B	0.9700
N1—C5	1.508 (4)	C5—C6	1.474 (5)
N1—H1C	0.9100	C5—H5A	0.9700
C1—C2	1.511 (5)	C5—H5B	0.9700
C1—H1A	0.9700	C6—C7	1.298 (5)
C1—H1B	0.9700	C6—H6A	0.9300
C2—H2A	0.9700	C7—H7A	0.9300
C2—H2B	0.9700	C7—H7B	0.9300
C3—C4	1.503 (5)		
			
C2—O1—C3	109.7 (3)	O1—C3—H3B	109.3
C4—N1—C1	109.1 (2)	C4—C3—H3B	109.3
C4—N1—C5	112.6 (2)	H3A—C3—H3B	108.0
C1—N1—C5	111.8 (2)	N1—C4—C3	109.7 (3)
C4—N1—H1C	107.7	N1—C4—H4A	109.7
C1—N1—H1C	107.7	C3—C4—H4A	109.7
C5—N1—H1C	107.7	N1—C4—H4B	109.7
N1—C1—C2	109.4 (3)	C3—C4—H4B	109.7
N1—C1—H1A	109.8	H4A—C4—H4B	108.2
C2—C1—H1A	109.8	C6—C5—N1	111.6 (3)
N1—C1—H1B	109.8	C6—C5—H5A	109.3
C2—C1—H1B	109.8	N1—C5—H5A	109.3
H1A—C1—H1B	108.2	C6—C5—H5B	109.3
O1—C2—C1	111.7 (3)	N1—C5—H5B	109.3
O1—C2—H2A	109.3	H5A—C5—H5B	108.0
C1—C2—H2A	109.3	C7—C6—C5	124.5 (4)
O1—C2—H2B	109.3	C7—C6—H6A	117.7
C1—C2—H2B	109.3	C5—C6—H6A	117.7
H2A—C2—H2B	107.9	C6—C7—H7A	120.0
O1—C3—C4	111.6 (3)	C6—C7—H7B	120.0
O1—C3—H3A	109.3	H7A—C7—H7B	120.0
C4—C3—H3A	109.3		
			
C4—N1—C1—C2	−55.2 (3)	C5—N1—C4—C3	−179.8 (3)
C5—N1—C1—C2	179.7 (3)	O1—C3—C4—N1	−58.9 (4)
C3—O1—C2—C1	−60.5 (4)	C4—N1—C5—C6	60.5 (3)
N1—C1—C2—O1	58.5 (4)	C1—N1—C5—C6	−176.4 (3)
C2—O1—C3—C4	60.7 (4)	N1—C5—C6—C7	−113.9 (4)
C1—N1—C4—C3	55.5 (3)		

### Hydrogen-bond geometry (Å, º)

**Table:**

*D*—H···*A*	*D*—H	H···*A*	*D*···*A*	*D*—H···*A*
N1—H1*C*···Br1^i^	0.91	2.31	3.218 (2)	175
C1—H1*A*···Br1^ii^	0.97	2.93	3.846 (4)	158
C5—H5*B*···Br1^iii^	0.97	2.86	3.796 (3)	162

Symmetry codes: (i) −*x*+1, −*y*+1, −*z*+1; (ii) *x*, *y*−1, *z*+1; (iii) −*x*, −*y*+1, −*z*+1.
